# Hemichannels in neurodegenerative diseases: is there a link to pathology?

**DOI:** 10.3389/fncel.2014.00242

**Published:** 2014-08-20

**Authors:** Megan Bosch, Tammy Kielian

**Affiliations:** ^1^Department of Pharmacology and Experimental Neuroscience, University of Nebraska Medical CenterOmaha, NE, USA; ^2^Department of Pathology and Microbiology, University of Nebraska Medical CenterOmaha, NE, USA

**Keywords:** connexin, hemichannels, neurodegeneration, lysosomal storage disease, Alzheimer’s disease

## Abstract

Although originally considered a structural component of gap junctions, connexin hemichannels (HCs) are now recognized as functional entities capable of influencing metabolic gradients within the CNS, allowing direct communication between the intra- and extracellular milieus. Besides connexins, HCs can also be formed by pannexins, which are not capable of gap junction assembly. Both positive and negative effects have been attributed to HC activity in the context of neurodegenerative diseases. For example, HCs can exert neuroprotective effects by promoting the uptake of neurotoxic molecules, whereas chronic HC opening can disrupt molecular gradients leading to cellular dysfunction and death. The latter scenario has been suggested for multiple neurodegenerative disorders, including Alzheimer’s disease (AD) and more recently, lysosomal storage disorders, which are the focus of this perspective. Currently available evidence suggests a complex role for HCs in neurodegenerative disorders, which sets the stage for future studies to determine whether targeting HC action may improve disease outcomes.

## Introduction

Hemichannels (HCs) are composed of six connexin (Cx) subunits that assemble into hexameric pores that traffic to the plasma membrane where they can remain uncoupled or pair with adjacent HCs on neighboring cells to form gap junction channels (Contreras et al., 2003). Besides Cxs, HCs can also be formed by pannexins (Panx), which are not capable of gap junction assembly. This Perspective focuses on HC involvement in neurodegenerative diseases; therefore, gap junction activity will not be discussed here but has been the subject of several excellent reviews related to CNS disorders (Orellana et al., 2009; Eugenin et al., 2012). HCs are permeable to small hydrophilic molecules, such as ATP, Ca^2+^, glutamate, glucose, and glutathione, which are critical for CNS homeostasis by maintaining ionic and metabolic gradients and can also control autocrine/paracrine signaling (Retamal et al., 2007; Kielian, 2008; Rouach et al., 2008; Sánchez et al., 2009; Schalper et al., 2010; Orellana et al., 2011a; Bennett et al., 2012; Fiori et al., 2014).

Currently, a total of 10 Cx and 2 Panx isoforms have been reported to be expressed in the brain (Giaume et al., 2013). The repertoire of Cx protein expression is distinct among various CNS cell types; however, the functional impact of these differences remains to be fully defined. For example, astrocytes primarily express Cx43 and Cx30, as well as, Cx26, Cx40, Cx45 and Cx46; microglia utilize Cx43, Cx36, and Cx32; and neurons express Cx36, Cx26, Cx45, and Cx57 (Rouach et al., 2002; Mika and Prochnow, 2012). Studies have shown that a selective pattern of Cx HC expression may orchestrate extracellular signaling networks between various glial cell types and/or neurons in a homotypic or heterotypic fashion (Giaume et al., 2013; May et al., 2013). Panx expression has been reported in neurons, astrocytes, and more recently microglia (Orellana et al., 2009, 2011b; Sáez et al., 2013b); however, an outstanding question is whether Cx/Panx HC function differs within various CNS cell subpopulations. For example, it is becoming clear that astrocytes exhibit regional heterogeneity and it will be interesting to determine whether this is associated with differences in the molecular composition of Cx/Panx HCs and/or sensitivity to HC opening.

**Figure 1:**
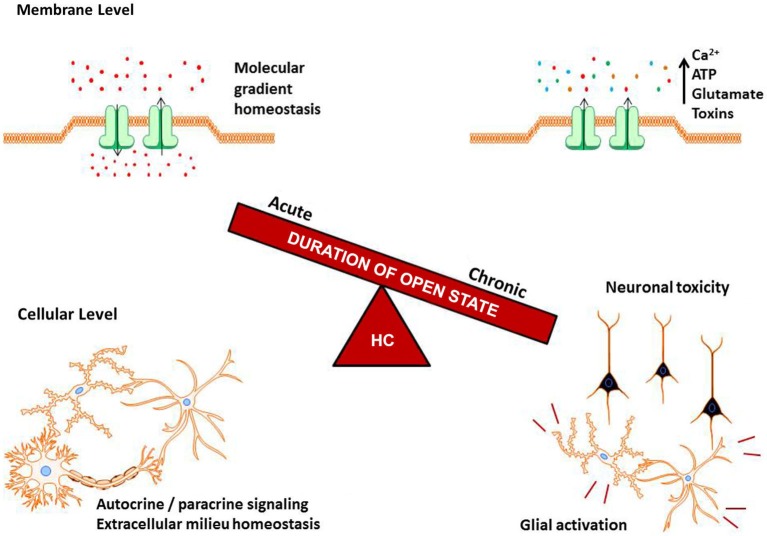
**Balance of hemichannel (HC) regulation in the CNS**. HC opening is under tight control, where acute activity helps to maintain molecular gradients, cell homeostasis, and autocrine/paracine signaling. A slight disruption of this balance, as can occur during neurodegenerative diseases and inflammatory signals, causes increased HC opening. Chronic HC activity can result in the ablation of vital molecular gradients, toxin release into the extracellular milieu, and potentiation of glial activation and neuron death.

Originally considered a structural component of gap junctions, strong evidence has emerged to support a role for HCs in maintaining cellular and tissue homeostasis, by allowing cells to directly communicate with their surrounding microenvironment and relay signals via the release of molecules that activate extracellular receptors in an autocrine/paracrine manner (Orellana et al., 2009). In the last decade, several reports have linked inflammation and dysregulated HC activity in the CNS. The summation of this work suggests that HCs have a dual role in regulating molecular gradient homeostasis in the context of neurodegenerative diseases. On the one hand, transient HC activity has been suggested to be protective during normal physiologic states as well as acute insults or inflammation. For example, astrocyte HCs have been shown to promote glucose uptake, which is considered a second glucose-lactate pathway for neurons. In addition, HCs could release lactate, which could also be beneficial for neurons (Rouach et al., 2008; Giaume et al., 2013). Proinflammatory cytokines (i.e., IL-1β and TNF-α) are known to activate glia and promote HC opening, which may serve as a means to propagate glial activation or neuron activity by the release of bioactive molecules acting in an autocrine/paracrine manner. Conversely, sustained HC opening during chronic neurodegenerative diseases may promote disease progression by perturbing metabolic gradients and the exaggerated release of toxic molecules to induce cell death (Figure 1).

## HC involvement in neurodegenerative diseases

A common denominator linking neurodegenerative diseases and HC opening is neuroinflammation, characterized by microglial and astrocyte activation and secretion of inflammatory mediators. Microglia, the resident macrophages of the CNS parenchyma, phagocytose cellular debris and produce a wide array of proinflammatory molecules (Kettenmann et al., 2011; Lyman et al., 2014). These mediators, which include cytokines (i.e., IL-β, IL-6, and TNF-α), reactive oxygen and nitrogen species, glutamate, and neurotrophic factors enhance cell mobility, phagocytosis, and can be neuroprotective when carefully regulated (Kielian, 2008; Mika and Prochnow, 2012). Under physiological conditions, transient astrocyte activation plays a pivotal role in maintaining neurotransmitter levels at the tripartite synapse and metabolite trafficking. However, during neuroinflammation, chronic proinflammatory mediator release may cause dramatic and potentially detrimental alterations in the way microglia and astrocytes communicate and regulate homeostasis via HC opening (Figure 1). The end result is a milieu that can lead to cellular toxicity or dysfunction, which over time, can manifest as cognitive and/or motor decline depending on the CNS site affected (Finn et al., 2011; Orellana et al., 2011a; Xiong and Kielian, 2013).

## HC activity in lysosomal storage diseases

Lysosomal storage diseases (LSDs) encompass a large group of inherited metabolic disorders characterized by the accumulation of storage material within lysosomes. Collectively, LSDs afflict 1 out of every 6700 live births and approximately 75% of the LSDs currently identified impact CNS function (Meikle et al., 1999; Sands and Haskins, 2008). Although a relatively new area of investigation, recent studies have reported perturbed HC activity in two distinct LSDs, namely Juvenile Neuronal Ceroid Lipofuscinosis (JNCL) and Niemann-Pick type C (NPC).

JNCL is caused by a mutation in the *CLN3* gene that most commonly spans exons 7–8 (Janes et al., 1996; Cotman et al., 2002; Drack et al., 2013). Brains of JNCL patients at autopsy as well as JNCL mouse models have shown that areas of activated microglia and astrocytes correlate with regions of neuron loss, along with elevated levels of IL-1β and ceramide, the latter representing a key lipid mediator involved in inflammation and apoptosis (Pontikis et al., 2004; Mencarelli and Martinez-Martinez, 2013; Xiong and Kielian, 2013). A recent study from our laboratory using primary microglia from the CLN3^Δex 7/8^ mouse model of JNCL demonstrated that when challenged with “danger signals” elevated in the brains of JNCL patients (i.e., ceramide and neuron lysate), CLN3^Δex 7/8^ microglia released significantly more proinflammatory mediators compared to wild type cells, which remained largely non-responsive (Xiong and Kielian, 2013). Furthermore, CLN3^Δex 7/8^ microglia displayed increased HC opening, which was associated with elevated glutamate and ATP release. Glutamate accumulation can cause neuronal excitotoxicity and a role for glutamate excitotoxicity in JNCL progression has been previously reported (Finn et al., 2011).

Another recent study from our laboratory revealed a transient increase in astrocyte HC activity in disease-affected regions of the CLN3^Δex 7/8^ mouse brain as early as postnatal day 30, which significantly preceded neuron loss that is not evident until 6–8 months of age (Burkovetskaya et al., 2014). However, this increase was transient, since CLN3^Δex 7/8^ astrocyte HC function began to decline at postnatal day 60, eventually falling below levels observed in wild type mice by postnatal day 90, suggesting a progressive decline in astrocyte function at later stages of disease. Treatment of CLN3^Δex 7/8^ mice with the HC inhibitor INI-0602, a blood-brain barrier permeable derivative of carbenoxolone (Takeuchi et al., 2011), significantly reduced lysosomal storage material accumulation in specific brain regions. In addition, astrocyte gap junction communication was significantly elevated in CLN3^Δex 7/8^ mice, which was predicted to occur via HC closure, although this was not apparent in acute brain slices *ex vivo* (Burkovetskaya et al., 2014). Nonetheless, aberrant HC activity in astrocytes and microglia may contribute to neuron loss in JNCL, particularly when considering that glial activation predates neuron death by several months in this mouse model (Pontikis et al., 2004). Unresolved questions are whether changes in HC function are responsible for the brain metabolic disturbances reported in JNCL and whether HC involvement extends to other CNS cell types (i.e., neurons and microglia).

NPC is caused by a mutation in the *NPC1* or *NPC2* genes, with the former being most common. NPC1 and NPC2 are required for cholesterol clearance and their absence causes the accumulation of cholesterol and other lipids in lysosomes (Rosenbaum and Maxfield, 2011). Similar to JNCL, neuroinflammation has been implicated in NPC pathology (Baudry et al., 2003). However, a recent study by Sáez et al. (2013a) suggested that the increase in HC activity observed in NPC might not be related to neuroinflammation *per se* but rather, the mutation itself (Sáez et al., 2013a). Specifically, primary astrocyte cultures from NPC^−/−^ mice displayed increased HC activity under baseline conditions compared with cells from wild type and NPC^+/−^ animals. In addition, acute hippocampal slices from NPC^−/−^ mice at postnatal day 2 revealed increased astrocyte HC activity that could be blocked using the general HC blocker La^3+^ and Cx43 antibody. These data imply the involvement of Cx43 HCs and suggest that dysfunctional HCs occur at the earliest phase of NPC disease. It remains to be determined whether this HC activity represents an attempt by astrocytes to regain homeostasis in the context of NPC mutation or whether HC opening sets the stage for downstream neuropathology. Nevertheless, the available evidence supports a role for HCs in two distinct LSDs that have devastating consequences on the CNS (Finn et al., 2011; Sáez et al., 2013a; Burkovetskaya et al., 2014).

## Alzheimer’s disease and HC function

Alzheimer’s disease (AD) is currently the leading cause of dementia in adults over the age of 65 years (Tiiman et al., 2013). Hallmark symptoms of AD include memory impairment, loss of abstract thought and language skills, and alterations in personality (Welander et al., 2009). Pathologically, AD is characterized by the extracellular accumulation of amyloid-beta (Aβ) peptide into senile plaques and the intracellular hyperphosphorylation of tau protein into neurofibrillary tangles. These aggregates cause neuronal damage, generation of reactive oxygen and nitrogen species, neuroinflammation, and defects in cell-cell communication (Selkoe, 2001; Small and Duff, 2008; Quintanilla et al., 2012; von Bernhardi and Eugenin, 2012). The underlying mechanisms that elicit plaque and tangle accumulation in AD remain elusive.

Using an APP/PS1 mouse model of AD, Mei et al. (2010) observed increased Cx43 and Cx30 expression in astrocyte processes invading the plaque core and Cx43 immunoreactivity has also been associated with plaques in human AD tissues (Nagy et al., 1996; Mei et al., 2010). Along with increased Cx expression, Aβ has been reported to increase HC activity in neurons, astrocytes, and microglia (Orellana et al., 2011b). Similar to the series of events reported in JNCL above, Aβ elicits a proinflammatory response in resident glial cells typified by inflammatory cytokine, glutamate, and ATP release, which subsequently triggers HC opening in neighboring neurons. It is postulated that neuron HC opening is one factor responsible for neuron death that can further enhance neuroinflammation and propagate the neurodegenerative process.

Indeed, HC involvement in AD was also demonstrated by Takeuchi et al. (2011), where treatment of APP/PS1 mice with the HC inhibitor INI-0602 improved cognitive function (Takeuchi et al., 2011). In addition, INI-0602 blocked neurotoxic glutamate release from activated microglia both *in vitro* and *in vivo*. Although still in the relatively early stage of exploration, these findings suggest that targeting HCs could prove to be beneficial in combating AD symptoms and progression.

## Current challenges in the field of HC biology

Since the discovery that HCs can exert functional activity, significant efforts have been made to characterize their roles in neurodegenerative diseases; however, many limitations still exist. One primary issue is the availability of reagents that can specifically block HC action. Many of the pharmacological inhibitors currently used to study HCs also affect gap junctions, which must be considered when these compounds are used experimentally. In addition, many widely used inhibitors are not selective for a particular HC type, which leaves the identification of HC protein composition in question. A potential solution is the use of Cx-specific antibodies or Cx/Panx mimetic peptides that can selectively block HC permeability (Sáez et al., 2013a). A second difficulty when studying Cx HCs is the methods used to evaluate their activity, since it is challenging to distinguish between Cx gap junction and HC action. For example, HCs are often reported by increases in either Cx immunoreactivity in brain tissues or Western blots. However, both gap junctions and HCs are comprised of Cxs making it problematic to discriminate between the two. Functionally, the primary method used to measure HC activity is ethidium uptake. However, based on its low molecular weight, numerous types of open channels are permeable to ethidium, which calls to caution how HC ethidium uptake data is interpreted. Here the combination of ethidium uptake coupled with a Cx/Panx selective inhibitor can begin to narrow the action to Cx/Panx channels; however, for the reasons mentioned above, identification of HC vs. gap junction channels remains an area of debate. Perhaps the best means to quantifying HC activity is single channel electrophysiology. This approach is feasible in cultured cells; however, it is significantly more challenging in brain slices, as reflected in a study by Kang et al. (2008) where electrophysiological evidence of HC activity was obtained in only 18 out of 700 recordings (Kang et al., 2008). Regardless of these technical limitations, careful consideration should be given to select the best model system to examine HC function. Many studies characterizing the functional roles of HCs have been performed in reconstituted liposomes or cell cultures (Fiori et al., 2014). While these methods provide useful insights, they are not able to recapitulate *in vivo* environments. Live tissue slices are better representative of *in vivo* events and have provided more functional data; however, this approach also has its limitations. Specifically, upon excision, the brain slice begins to slowly deteriorate due to cell damage inflicted during the cutting process concomitant with microglial activation, which may impact the results obtained. To ensure tissue viability for an extended period of time, slices are continuously bathed with artificial cerebrospinal fluid which is equilibrated with CO_2_ and care should be taken to evaluate cells that are positioned well below the cut surface to avoid signals from damaged regions. Future research avenues could be directed towards discovering new minimally invasive methods for studying HCs *in vivo* such as two-photon microscopy or channel tracers that can be imaged using functional magnetic resonance imaging (MRI) or single photon emission computed topography (SPECT; Kielian, 2008).

## Outstanding questions and conclusions

Significant progress has been made towards elucidating the functional role of HCs in neurodegenerative diseases; however, many unanswered questions remain. The first is whether HC dysfunction is an active contributor to disease pathology or merely a consequence. In the case of AD, neuroinflammation indirectly increases HC channel activity in multiple cell types (Orellana et al., 2011a; Quintanilla et al., 2012). Numerous reports by others also support a similar relationship where HC activation and neuroinflammation act as co-factors, where one event influences the progression of the other. If correct, then therapeutically targeting HCs would only potentially slow disease progression but not be curative. In contrast, a direct connection between HC activity and inflammation does not appear to exist in NPC. Specifically, increased HC activity in neurons, astrocytes, and microglia was not induced by proinflammatory stimulators, suggesting that HC action precedes neuroinflammation in NPC. A similar case for changes in HC activity predating neuroinflammation is seen in JNCL, since HC dysfunction was observed early, whereas overt inflammation is not evident until later stages of disease (Xiong and Kielian, 2013; Burkovetskaya et al., 2014). The similarities between these two LSDs suggest that a core response may be operative that is not tied to inflammation *per se*. Nonetheless, as the intensity of the inflammatory response increases with advancing disease, it is likely that inflammatory mediators will impact HC activity, perhaps in a fashion that has already been described in AD and glial cell culture models (Figure 1). The complexity of HC composition combined with distinct expression patterns on various CNS cell types suggest that new insights are bound to emerge to account for this diversity in HCs in terms of CNS homeostasis and pathology.

## Conflict of interest statement

The authors declare that the research was conducted in the absence of any commercial or financial relationships that could be construed as a potential conflict of interest.
